# A cognitive accessibility review of national palliative care resources with people with cognitive disabilities

**DOI:** 10.1186/s12904-026-02067-3

**Published:** 2026-03-14

**Authors:** Niima Er-raji, Munazza Tahir, Lynn Martin

**Affiliations:** 1 Open Collaboration for Cognitive Accessibility, University of Ottawa, Ottawa, Canada; 2 Department of Health Sciences, Lakehead University and Centre for Education and Research on Aging and Health, Lakehead University, Thunder Bay, Canada

**Keywords:** Cognitive accessibility, Developmental disabilities, Dementia, Palliative care, Patient and public involvement, Inclusive design

## Abstract

**Background:**

People with cognitive disabilities (including developmental disabilities and dementia) face significant inequities in accessing palliative care information and services. Accessible communication plays a critical role in enabling meaningful participation in care, yet few national palliative care resources are evaluated with direct input from people with lived experience.

**Methods:**

A qualitative descriptive study engaged eight individuals with lived experience of cognitive disabilities as cognitive accessibility experts. These individuals reviewed three palliative care resources (in English or French) and participated in semi structured virtual interviews. Data were analyzed to identify accessibility challenges, strengths of resources, and actionable recommendations.

**Results:**

The analysis revealed challenges such as dense vocabulary, abstract phrasing, and inconsistent sequencing. At the same time, cognitive accessibility experts by experience identified strengths, including plain-language sections, clear segmentation, simple visual layouts, and useful questions to ask healthcare providers. In particular, their comments praised the organization, straightforwardness, and readability. Based on these insights, cognitive accessibility experts by experience recommended simplifying language, improving clarity and structure, adding concrete examples, and incorporating more direct communication prompts. Health Canada has already begun revising the resources in response to these recommendations, demonstrating real-world impact.

**Conclusions:**

Cognitive accessibility depends on how the information is designed, not on the person reading it. Involving people with cognitive disabilities as research partners strengthens both the ethical foundation and practical utility of palliative care communication. Co-designed, cognitively accessible resources are essential to support autonomy, shared decision-making, and equitable palliative care access for all.

**Supplementary Information:**

The online version contains supplementary material available at 10.1186/s12904-026-02067-3.

## Background

Cognitive disabilities can be functionally defined by challenges in attention, memory, learning, and social comprehension [1]. Individuals with cognitive disabilities may have lived experience with intellectual or developmental disabilities as well as those living with dementia. Globally, developmental disabilities affect an estimated 2–3% of the population, representing approximately 160 to 240 million people worldwide [2, 3], and dementia currently affects 55 million people [4]; dementia is also much more prevalent among persons with developmental disabilities, and those with Down syndrome in particular [5]. In this context, accessibility refers to the design of environments, information, and systems in ways that enable inclusion and meaningful participation for individuals with diverse abilities [6]. Within digital and health information contexts, cognitive accessibility constitutes a core component of comprehensive accessibility and focuses on ensuring that information and services are understandable, intuitive, and usable for individuals with cognitive disabilities [7].

Persons with cognitive disabilities experience a heightened risk of medical comorbidities due to associated syndromes, secondary conditions, and a risk of late detection because of communication barriers [8]. For example, studies show that adults with intellectual or developmental disabilities exhibit much higher rates of chronic health conditions [9], as well as high multimorbidity at younger ages [10]. Similarly, more than half of people living with dementia have comorbidity at the time of diagnosis, and dementia itself is associated with markedly increased mortality [11, 12]. Comorbidity also significantly reduces quality of life in people with dementia, further underscoring the relevance of palliative approaches for this group [13].

As such, there is a strong imperative to adopt an accessible palliative care approach that enhances quality of life by attending not just to physical symptoms but also psychosocial and spiritual dimensions and honors the person’s care preferences [14]. However, systemic barriers, including healthcare providers’ biases, discomfort talking about death, and a lack of tailored resources, prevent this large and growing population from receiving equitable services [15, 16]. Emerging evidence highlights that persons with developmental disabilities [15, 17] and individuals living with dementia [18] remain underserved in palliative care, particularly due to gaps in accessibility that limit equitable communication, symptom management, and participation in care planning.

For palliative care to be effective for people with cognitive disabilities, it must be tailored to address both their medical complexity and their specific cognitive accessibility needs [19]. Research confirms that with appropriate support, people with cognitive disabilities are experts in their own needs and can effectively express their preferences, emphasizing a strong preference for direct communication and involvement in their own care [20, 21]. A collaborative model of decision-making, involving the individual, their family, and professionals, is therefore essential to balance autonomy with necessary support, ensuring timely and person-centered care that respects their rights and preferences [20–22].

There are several global, national, and local organizations that have developed palliative care resources to support persons with life-limiting illnesses. For example, the WHO has released resources that explain what palliative care is, who it is for, and how it is provided [23]. However, there is a lack of palliative care resources that are cognitively accessible and designed for people with cognitive disabilities themselves, in spite of laws like the United Nations Convention on the Rights of Persons with Disabilities [24] or the Accessible Canada Act [25] which mandate the provision of accessible information formats. A review of the literature identifies a significant research gap, with existing studies narrowly focused on intellectual disabilities and neglecting the broader spectrum of cognitive conditions. Only one study, conducted in the UK has developed a resource for professional caregivers using established criteria for accessible and effective information booklets [26]. Critically, the involvement of people with lived experience in the design of these resources is virtually absent, undermining their autonomy and leading to resources that are likely much less useful.

To address these critical and persistent gaps, the present study conducts an evaluation of governmental resources on palliative care. This research is specifically justified by the lack of Canadian studies that evaluate resources designed for individuals with cognitive disabilities, despite the legal mandate for accessibility. This study utilized an inclusive approach with direct engagement of individuals with lived experience of cognitive disabilities, thereby addressing a fundamental gap in existing literature. The study aimed to describe the experiences of individuals with cognitive disabilities with palliative care resources and to identify co-designed modifications needed to make these resources cognitively accessible.

## Methods

Ethical approval was granted by the institutional Research Ethics Board at the University of Ottawa.

### Study design and inclusive approach

This study employed a qualitative description approach [27] to evaluate the cognitive accessibility of three palliative care resources. The evaluation was conducted in partnership with Open Collaboration for Cognitive Accessibility (Open), which brings together experts and people with diverse cognitive abilities to co-design accessible products and build an inclusive research community [28]. The study centered on the insights of individuals with lived experience of cognitive disabilities, who were paid for their work. In this paper, participants are referred to as cognitive accessibility experts by experience. A semi structured interview format was used to gather detailed feedback on their perspectives on accessing, navigating, and understanding the resources. The interviews were 30 min long. This study incorporated the perspectives and experiences of the intended users, aligning with principles of inclusive and participatory research.

### Recruitment

A total of eight cognitive accessibility experts by experience (*n* = 8) were recruited for this study by the engagement team at Open via an initial email, although nine cognitive accessibility experts had originally been targeted for recruitment. The recruitment goal was to allow for a balanced representation of linguistic backgrounds, ideally including three bilingual cognitive accessibility experts, three French-speaking cognitive accessibility experts, and three English-speaking cognitive accessibility experts.

Individuals were eligible to participate if they were over 18 years of age. Cognitive accessibility experts by experience and could understand and communicate in at least one official language (English or French); who expressed interest were subsequently sent a participation email with further instructions. The final cohort of cognitive accessibility experts by experience consisted of three English-speaking, two French-speaking, and three bilingual individuals. The eight cognitive accessibility experts by experience ranged in age from 20 to 48 years and included six women and two men from several Canadian cities. Cognitive accessibility experts reported diverse lived experiences including autism, ADHD (Attention Deficit and Hyperactivity Disorder), intellectual and developmental disability, stroke-related physical disabilities, PTSD (Post-Traumatic Stress Disorder), and learning difficulties. Cognitive accessibility experts by experience reported diverse ethnic backgrounds, including Caucasian, Northern European and Scandinavian heritage, North African and Middle Eastern backgrounds, while some experts did not specify their ethnicity.

### Resources

The resources evaluated included three PDF documents developed by Health Canada in 2024, available in English and French: (1) a two-page fact sheet on communicating with a healthcare team after a serious illness diagnosis (Resource 1: “What to do when facing a serious illness?” / “Quoi faire face à une maladie grave ?“), (2) a poster summarizing how a palliative care approach can improve quality of life (Resource 2: “Living with a serious illness” / “Vous avez une maladie grave”), and (3) an infographic describing palliative care, clarifying misconceptions, and distinguishing between a palliative approach and specialist palliative care (Resource 3: “Explore the full spectrum of palliative care” / “Explorez la gamme complète des soins palliatifs”).

### Procedure

Cognitive accessibility experts by experience were sent the three PDF documents via email along with instructions. Each reviewed resources in their preferred language; those who were bilingual reviewed all resources. Once they had reviewed the resources, a virtual interview (using Microsoft Teams) was scheduled with the Research Coordinator using a Calendly page. Cognitive accessibility experts by experience were offered the option to receive the interview questions one week in advance and to be accompanied by a support person (e.g., a caregiver, family member, or friend) during the meeting. Having a support person present was not required.

At the beginning of each meeting, the interviewer read a consent script and obtained verbal consent before audio-recording the meeting. Semi-structured interviews were conducted using an interview guide (see Table S1 in Supplementary Materials) to ensure consistent topics were covered while allowing for flexibility to explore individual responses. Each was asked about their experience with technical aspects (e.g., opening and navigating the PDFs) and cognitive aspects (e.g., understanding the language and structure) of the resources.

### Analysis

A qualitative descriptive approach was used to summarize the cognitive accessibility expert interviews, documenting participants’ experiences exactly as they described them [27]. Feedback was then compiled and organized into a single Excel matrix (see Table 2 in Appendices). This matrix facilitated the thematic organization of feedback for each resource, allowing the identification of common challenges, points of satisfaction, and specific recommendations for improving the cognitive accessibility of the different resources. The Excel matrix (see Table 2 in Appendices) organized Cognitive accessibility experts’ responses into structured columns reflecting important elements of the interview guide, including demographic information, technical accessibility (e.g., opening the PDF, scrolling, device used), visual and cognitive accessibility (e.g., ability to stay focused, visual comfort, understanding of the text), and comprehension of palliative care concepts. It also included columns documenting words that were easy or difficult to understand, cognitive accessibility experts’ interpretations of palliative care, and detailed feedback for each resource. This structure facilitated comparison across cognitive accessibility experts, helping identify recurring patterns in the feedback and supporting the development of themes and recommendations regarding the cognitive accessibility of the resources.

The findings were synthesized to highlight key takeaways regarding the overall accessibility of the resources.

### Post-review language adaptation

Following the cognitive accessibility review, the resources were adapted by a language specialist using ASC (Accessibility Standards Canada) Plain Language and Easy-Read guidelines [29]. These adaptations were informed by the findings generated through interviews with cognitive accessibility experts by experience. The purpose of this step was to improve cognitive accessibility while preserving the original meaning and intent of the materials. The adaptation process focused on simplifying sentence structure, clarifying vocabulary, strengthening information hierarchy, and making action steps more explicit in both English and French versions of the resources.

## Results

The qualitative review of the three Health Canada informational resources on palliative care revealed some variability in cognitive accessibility as perceived by cognitive accessibility experts by experience (*n* = 8). While they recognized the importance of the material, they noted some challenges with linguistic complexity, conceptual ambiguity, and structural organization.

### Cognitive accessibility evaluation with cognitive accessibility experts by experience

#### Resource 1: what to do when facing a serious illness?

Cognitive accessibility experts by experience expressed mixed impressions of this resource. Several cognitive accessibility experts by experience found the inclusion of guiding questions and the sectioning of content to be constructive. The segmentation of topics into boxed questions provided visual relief and supported comprehension. As one cognitive accessibility expert explained, “*I liked that each question was in its own rectangle*,* it made it less overwhelming*” (Willy). Another cognitive accessibility expert valued its practical orientation: “*I would use these questions*” (Diana). However, others reported that the text was “too busy” and overwhelming due to its length and some abstract vocabulary. One cognitive accessibility expert noted, “*I had to read it a few times to understand*,* it’s a wall of text*” (Lana). Specific terminology was flagged as overly formal or confusing, creating a barrier to understanding.

#### Resource 2: living with a serious illness

This resource was consistently described as the most cognitively accessible. Cognitive accessibility experts by experience highlighted its concision, clear structure, adherence to plain language, and visually simple layout. One cognitive accessibility expert shared, “*It was clear and easy to read. I didn’t have any trouble with it*” (Rita). Key design features, such as large print, bullet points, and short sentences, supported readability. No notable barriers were reported for the English version; only minor linguistic simplifications, such as using simpler terms: “accès à du soutien”(access to support) to “obtenir de l’aide” (get help), “tous les stades de la maladie” (all stages of the illness) to “toutes les étapes de la maladie” (all steps/phases of the illness), and “fournisseur de soin” (care provider) to “docteur” (doctor) to improve clarity.

#### Resource 3: explore the full spectrum of palliative care

Cognitive accessibility experts by experience described this document as the most difficult to understand. Many needed multiple readings to grasp its terminology and conceptual distinctions. One cognitive accessibility expert remarked, “*I had to read it a few times; the sentences were long and run together*” (Willy). Another shared confusion about the repeated use of similar terms: “*The difference between the palliative approach and specialist palliative care was hard to understand when they were used right after each other*” (Diana). Further, repetition, particularly the frequent appearance of “care,” hindered comprehension. A French-speaking cognitive accessibility expert similarly noted, “*Les mots ‘soins palliatifs’ et ‘soins palliatifs spécialisés’ mis l’un après l’autre*,* c’était difficile à suivre*’’ (English translation: *The words ‘palliative care’ and ‘specialized palliative care’ placed one after the other were difficult to follow*) (Hiba).

Most advisors found the resource helpful and appreciated features such as its organization and web links; however, many also noted opportunities to strengthen linguistic clarity and structural presentation.

### Cross-resource findings

Across all documents, most cognitive accessibility experts found opening and scrolling through PDFs straightforward using personal computers, smartphones, or tablets. The simple visual presentation, pastel color schemes, black text, and supportive imagery, was perceived positively. One cognitive accessibility expert stated, “*The colors were soft on my eyes*,* and the text was spaced well*” (Leo).

Despite these strengths, many noted some challenges with dense paragraphs, long sentences, and vocabulary. Some terminology (e.g., “generalist,” “optimiste (which translates in English to : *optimistic*)” “précoce (which translates in English to : *precocious*),” “accablant (which is a more complex version of the word in English: *overwhelming*)”) was difficult to understand. A bilingual cognitive accessibility expert described switching languages to support comprehension: “*Sometimes I read in English to go faster*,* and other times I read in French to understand the terms*” (Hiba).

Confusion also arose when key definitions appeared only later in the text, or when visual markers such as arrows were used in non-sequential ways (e.g., functioning as bullet points rather than indicating a process), reducing clarity.

### Findings from the language specialist review and adaptations

A language specialist reviewed and adapted the original Health Canada palliative care resources using ASC Plain Language and Easy-Read guidelines, with the goal of making the information easier to understand and less intimidating for people living with serious illness and their caregivers. While the original materials were accurate and comprehensive, the adaptations focused on reducing the effort required to read and process the information, especially during times of stress.

Several meaningful changes emerged through the adaptation process:

- Core ideas were simplified and repeated so readers did not have to remember complex explanations.

*For example*, the idea that palliative care can help at any stage of illness was restated in clear, simple language rather than explained once and assumed to be understood.

- Sentences were shortened and separated, with one idea presented at a time.

*For example*, long paragraphs describing how palliative care supports people were broken into short lines or bullet points that are easier to scan and reread.

- Abstract or technical language was replaced with everyday words.

*For example*, phrases like “general pattern of illness” or “decision points” were rewritten as direct questions such as “What can I expect?” or “What happens next?”

- Important information was made easier to find.

*For example*, key messages about what palliative care is and how it helps were placed under clear headings and bullet points, rather than embedded within longer explanations.

- Emotions were named more openly and simply.

*For example*, grief was defined in plain terms (“Grief is a feeling of great sadness”), rather than being mentioned briefly or indirectly.

- Actions were made clear and visible.

*For example*, prompts such as “Talk to your health care provider” or “Ask your health care team these questions” were presented on their own lines instead of being buried in paragraphs.

## Discussion

This study examined the cognitive accessibility of three palliative care resources created, drawing directly on the perspectives of individuals with lived experience of cognitive disabilities. Overall, the most common challenges across resources included use of complex or abstract terminology; dense text and lengthy sentence structures; inconsistent sequencing of explanations and definitions; and insufficient examples to support understanding.

The findings showed variation across the three documents, with recurring challenges related to dense vocabulary, abstract terms, and uneven sequencing. At the same time, participants identified several strengths that supported comprehension, including clear segmentation, boxed questions, plain language, and simple layouts. Some cognitive accessibility experts by experience described certain resources as easy to read, well organized, and straightforward. One highlighted the usefulness of having specific questions to ask healthcare providers, noting that such prompts made difficult conversations more manageable. Similarly, the adaptations by the language specialist did not change the meaning of the information but changed how approachable it felt. By simplifying language, acknowledging emotions, and clearly guiding readers on what to do next. These strengths indicate that the resources form a solid foundation for accessible palliative communication. Based on these findings, key recommendations included (1) simplifying vocabulary, (2) shortening sentences, (3) introducing definitions earlier in the health resource, (4) using consistent visual cues, (5) reducing conceptual repetition, (6) adding concrete examples, (7) expanding direct questions and conversation prompts, and (8) keeping text density manageable. Together, these insights show that cognitive accessibility depends on intentional design choices, and that thoughtful communication practices can reduce barriers while enhancing the overall effectiveness of palliative care resources. It is also important to note that these recommendations would not have been possible without the direct involvement of persons with lived experience of cognitive disabilities.

For people with developmental disabilities and for those living with dementia, cognitively accessible information is essential to receiving equitable palliative care. Research has long shown that these individuals face substantial disparities in symptom recognition, communication, and decision-making, often because systems are not structured to meet their accessibility needs [30]. High rates of multimorbidity further increase the need for clear, supportive information to guide planning and participation in care [13]. The present findings illustrate how inaccessible language, unclear definitions, and conceptual overload can undermine a person’s ability to engage meaningfully in palliative discussions, reinforcing long-standing inequities.

The barriers identified across these documents underscore the need for palliative resources that are co-designed with the people who use them. Health literacy research highlight the importance of plain language, logical sequencing, concrete examples, reduced jargon, and visual clarity [31]. These were reflected in the feedback of the cognitive accessibility experts by experience (i.e., the need for clearer definitions, shorter sentences, consistent visual markers, bilingual clarity, and accessible formats beyond PDFs). These findings align with accessibility requirements in Canadian law, including the Accessible Canada Act [25], and point toward practical steps for improving national palliative communication.

The study’s findings reinforce the requirement for accessible health information under the Accessible Canada Act and the UNCRPD. Cognitive accessibility is also foundational to core components of palliative care such as shared decision-making, advance care planning, and maintaining autonomy [32]. Clear, understandable resources are therefore essential for ethical, rights-based, person-centered care and for reducing disparities that disproportionately affect people with cognitive disabilities.

An important contribution this study makes is its approach to patient and public involvement. Individuals with cognitive disabilities participated as paid cognitive accessibility experts by experience and provided detailed, experience-based insights that could not be gathered solely through traditional researcher-led evaluation. Their involvement aligns with growing evidence that meaningful participation enhances both ethical integrity and scientific quality in palliative care research [33, 34]. International frameworks such as the UN Convention on the Rights of Persons with Disabilities [24] affirm the right to accessible health information; yet people with cognitive disabilities remain excluded from many stages of research and resource development. This study demonstrates the feasibility and value of addressing that gap directly.

In addition to being informed by lived experience, other strengths of the study include its bilingual scope and focus on nationally distributed resources with broad public reach. In fact, the recommendations of this cognitive accessibility evaluation have already been put into action in a rapid uptake process resulting in revisions to nationally distributed resources [35–37]. This rapid uptake shows the practical value of involving people with cognitive disabilities in evaluating public-facing health information and demonstrates the potential for national systems to enact change when accessibility issues are clearly identified.

Limitations of this study include the modest sample size typical of qualitative patient and public involvement studies, the focus on PDF formats, and the absence of evaluation across multiple media or clinical settings. Future work could broaden the range of resources studied and explore accessibility in real-life care interactions. It should also take into account the views and experiences of a more diverse sample of persons with lived experience of cognitive impairments.

Further research should expand co-design approaches, develop Easy Read and multimedia versions of palliative resources, and assess how accessible communication influences decision-making, care engagement, and patient outcomes. Additional work with caregivers, clinicians, and culturally diverse communities would help refine accessibility strategies and ensure that palliative care reaches people equitably across contexts.

## Conclusion

This study highlights significant inconsistencies in the cognitive accessibility of federal palliative care information and demonstrates the importance of involving individuals with cognitive disabilities in evaluating and improving these resources. By centering lived experience, the findings offer concrete strategies for enhancing information design and strengthening equitable access to palliative care. Cognitive accessibility is essential not only for communication but for dignity, autonomy, and meaningful participation in care.

## Supplementary Information

Supplementary Material 1.

## Data Availability

The data and materials that support the findings of this study are not openly available due to reasons of sensitivity and are available from the corresponding author upon reasonable request.
