# Supplementary material for: A cognitive accessibility review of national palliative care resources with people with cognitive disabilities
Source: BMC Palliat Care. 2026 Mar 14;25:106. doi: 10.1186/s12904-026-02067-3 (PMC13101393; doi:10.1186/s12904-026-02067-3)
Supplement: Supplementary file 1 — Supplementary Material 1. [file 12904_2026_2067_MOESM1_ESM.docx]

# **Table 1. Interview guide**

| **Consent and intro**    Cognitive accessibility experts by experience were informed that the study focused on palliative care, described in simple terms as extra support for people living with serious illnesses that cannot be cured. They were told they would be invited to share their opinions on easy-to-understand documents on palliative care. The interviewer explained that the interview would be recorded, securely stored using password protection, accessed only by the research team, and deleted once the study was completed.  Participants were reminded in a supportive way that they could pause, take breaks, stop the interview at any time, or ask questions if needed. The interviewer also expressed appreciation for their contribution to improving the accessibility of palliative care resources and confirmed that they felt comfortable before beginning the conversation.      **Background questions (demographics)**   - The questions asked were about age, gender, city of origin, spoken, languages, and ethnic background.       **Main questions (PDF Document)**  *Example of technical questions*   - How easy was it for you to open the PDF files?**Easy, medium, or hard**? Why? - How easy was it for you to scroll through the file? **Easy, medium, or hard**? Why? - How easy was it for you to stay focused while reading the document? **Easy, medium or hard**? And what made it **Easy, medium or hard**for you to stay focused?     Example of questions about language accessibility and palliative care   - How easy was it to understand the text?**Easy, Medium or Hard**? - Which words do you find hard to understand? Why? - After reading the document, what do you think palliative care is? - What is something new that you learnt from reading this document? |
| --- |

**Table 2 –Template of the analysis matrix (rows and columns are transposed here to improve readability within the document layout).**

| **Participant ID** | **Participant P1** | **Participant P2** |
| --- | --- | --- |
| Disability / Lived Experience |  |  |
| Age |  |  |
| Gender |  |  |
| City |  |  |
| Language |  |  |
| Ethnicity |  |  |
| Ability to Open PDF |  |  |
| Device Used |  |  |
| Ability to Scroll Through File |  |  |
| Ability to Stay Focused |  |  |
| Visual Comfort (text size, colors, layout) |  |  |
| Ability to Understand Text |  |  |
| Words Easy to Understand |  |  |
| Words Difficult to Understand |  |  |
| Understanding of Palliative Care |  |  |
| Who Palliative Care Is For |  |  |
| What Participant Learned |  |  |
| Additional Comments |  |  |
| Feedback –  What to Do When Facing a Serious Illness |  |  |
| Feedback –  Living with a Serious Illness |  |  |
| Feedback –  Explore the Full Spectrum of Palliative Care |  |  |
